# Screening of Single-Domain Antibodies to Adeno-Associated Viruses with Cross-Serotype Specificity and a Wide pH Tolerance

**DOI:** 10.3390/v17101289

**Published:** 2025-09-23

**Authors:** Hailing Guo, Shuo Wang, Lujin Feng, Weiwei Xu, Jiandong Zhang, Xiaoju Zhou, Ningning Ma

**Affiliations:** 1 Wuya College of Innovation, Shenyang Pharmaceutical University, Shenyang 110016, China; ghling0906@163.com (H.G.); wangshuo_0204@163.com (S.W.); f15662002002@163.com (L.F.); 2 YSK Bio Co., Ltd., Zhuji 311899, China; 15027507162@163.com (W.X.); jiandong.zhang@vbiosci.com (J.Z.); 3 School of Life Science and Biopharmaceutics, Shenyang Pharmaceutical University, Shenyang 110016, China

**Keywords:** AAV, VHH, competitive biopanning strategy, alkaline stability, affinity purification

## Abstract

Adeno-associated virus (AAV) vectors are the preferred gene delivery tool in gene therapy owing to their safety, long-term gene expression, broad tissue tropism, and low immunogenicity. Affinity ligands that can bind multiple AAV serotypes endure harsh clean-in-place (CIP) conditions and are critical for industrial-scale purification. However, current ligands lack broad serotype recognition and adequate alkaline stability, which limits their reusability in large-scale manufacturing. In this study, we employed a competitive biopanning strategy to isolate a single-domain antibody (VHH) that simultaneously binds AAV2, AAV8, and AAV9. The VHH retained structural integrity and binding activity after exposure to 0.1 M NaOH, demonstrating robust alkaline stability. Structural modeling revealed that the VHH primarily recognizes the DE loop region of the VP3 capsid protein across the three serotypes, explaining its cross-serotype reactivity. Affinity chromatography using the VHH yielded infectious AAV particles, confirming its potential for downstream processing. This strategy provides a versatile platform for developing high-performance AAV affinity ligands and may be extended to other viral vector systems.

## 1. Introduction

Adeno-associated virus (AAV) has emerged as a pivotal vector in gene therapy owing to its low immunogenicity, non-pathogenic nature, and capacity for sustained transgene expression [1,2,3]. Since its initial discovery in 1965, 13 naturally occurring serotypes and numerous engineered variants have been identified, each exhibiting distinct tissue tropism profiles and therapeutic potential [4]. To date, several AAV-based gene therapies have received regulatory approval, underscoring the clinical potential of this platform. Representative examples include Luxturna (AAV2) for RPE65-mediated retinal dystrophy, Zolgensma (AAV9) for spinal muscular atrophy, Roctavian (AAV5) for hemophilia A, Hemgenix/Beqvez (AAV5) for hemophilia B, and Elevidys (AAV-rh74) for Duchenne muscular dystrophy [5,6,7,8,9,10]. Despite these advances, the utility of natural AAV serotypes is limited by restricted tissue tropism, widespread pre-existing immunity, and suboptimal transduction efficiency, thereby driving the development of engineered capsids with enhanced specificity and efficacy [7,9,10]. For example, AAV-DJ exhibits broad tissue tropism [11,12]; AAV-PHP.B and AAV-PHP.eB markedly enhance CNS delivery in murine models [13]; AAV-LK03 demonstrates exceptional liver specificity [14,15]; Anc80L65 efficiently targets the cochlea, retina, and liver [16,17,18]; and AAV2.7m8 has been optimized for targeted delivery to retinal photoreceptors [19].

Advancing AAV therapeutics requires robust manufacturing, with affinity chromatography serving as the preferred purification strategy for its selectivity, scalability, and good manufacturing practice (GMP) compliance [20]. Current research on affinity ligands for multi-serotype AAV purification has primarily focused on three strategic approaches: (1) VHH-based ligands [21,22], such as POROS CaptureSelect AAV8, AAV9, and AAVX resins (Thermo Fisher Scientific, Waltham, MA, USA), as well as AVB-Sepharose (GE Healthcare, Chicago, IL, USA), are derived from VHH fragments. (2) Peptide ligands [23,24], such as AVIPure AAV Affinity Resins (Repligen Corporation, Waltham, MA, USA). These ligands obtained through combinatorial screening or computer-aided design, often adopt cyclic or conformationally constrained structures. (3) Engineering and combinatorial strategies, including the design of “consensus” binding domains informed by structural biology [20], or the introduction of specific ligand-binding epitopes into the AAV capsid via protein engineering [25]. While various strategies offer distinct advantages, no ligand has yet achieved optimal affinity, and process compatibility for a given serotype. Among them, VHH ligands stand out for their stability, low immunogenicity, and strong binding affinity [26,27]. Their defined origins and amenability to rational engineering further support structural robustness under harsh conditions, enabling both laboratory and large-scale applications [28].

The construction of engineered AAV capsids often relies on the modification of natural serotypes; therefore, addressing the purification challenges of natural serotypes in large-scale production is not only of direct practical significance but also provides a methodological foundation for future process development of engineered capsids. Among the numerous AAV serotypes, AAV2, AAV8, and AAV9 are not only the most widely utilized in clinical trials and approved gene therapy products, but also exhibit marked differences in tissue tropism, capsid architecture, and binding characteristics with existing affinity ligands [29]. These features make them representative models for evaluating the broad-spectrum performance of affinity ligands. Furthermore, the three serotypes encompass diverse delivery strategies—from conventional tissue-targeted delivery to the ability of crossing the blood–brain barrier—where purification efficiency directly influences both clinical outcomes and large-scale manufacturing processes.

To address the limitations of prolonged screening cycles and the difficulty in balancing affinity with serotype adaptability for VHH ligands, our study incorporated a competitive biopanning strategy into the phage display workflow to preferentially isolate VHHs capable of binding AAV2, AAV8, and AAV9. This approach improved cross-serotype compatibility, although variations in binding affinity remained to be optimized. The identified VHH exhibited excellent alkaline stability, retaining activity after exposure to 0.1 M NaOH, thereby fulfilling the cleaning-in-place (CIP) requirements of GMP-compliant processes. This property facilitates the long-term reuse of affinity resins and supports industrial-scale manufacturing. Based on these findings, we developed and validated AAV2/8/9-specific VHH affinity ligands, offering a new solution for high-purity, scalable, and process-compatible AAV production.

## 2. Materials and Methods

### 2.1. Materials

Adeno-associated virus serotypes 2, 8, and 9 (AAV2, AAV8, AAV9) were purchased from PackGene Biotech Co., Ltd. (Guangzhou, China). A phage-displayed immune VHH library was provided by YSK Bio Co., Ltd. (Zhuji, China), which contracted Shenzhen KangTi Biopharm Technology Co., Ltd.(Shenzhen, China) immunizing a single alpaca with AAV2, AAV8, AAV9. Mouse monoclonal anti-M13 antibody was obtained from AlpVHHs™ (Chengdu, China), and mouse monoclonal anti-His antibody was sourced from Proteintech Group, Inc. (Wuhan, China). The MonoRab™ AAVX VP1/VP2/VP3 antibody (clone 5G4) was supplied by GenScript Biotech Corp. (Nanjing, China). 293F cells and their complete culture medium, 293T cells, Ni-NTA affinity resin, and CNBr-activated Sepharose 4 FF were all procured from YSK Bio Co., Ltd. (Zhuji, China).

### 2.2. Competitive Biopanning Strategy

AAV9 and AAV8 viral particles were diluted in phosphate-buffered saline (PBS, Solarbio, Beijing) to a final concentration of 1 × 10^9^ VP/mL and immobilized in 96-well plates at 4 °C overnight. The following day, wells were washed three times with PBST (PBS supplemented with 10% Tween-20, Solarbio, Beijing) and blocked with 3% bovine serum albumin (BSA, Genview, Houston, TX, USA) at 37 °C for 2 h. In parallel, 100 μL of the phage display library solution was mixed with 900 μL of 3% BSA and was incubated at 37 °C for 2 h for pre-blocking in order to eliminate VHHs that bind to BSA.

After blocking, wells were washed three times with PBST and incubated with the pre-blocked phage library in AAV9-coated wells at 37 °C for 1 h. Non-specifically bound phages were removed by ten washes with PBST. Bound phages were then competitively eluted by incubation with AAV2 viral particles (1 × 10^9^ VP/mL) at room temperature for 30 min. The eluted phages were transferred to AAV8-coated wells and incubated at 37 °C for 1 h. Following ten washes with PBST, specifically bound phages were eluted with 100 μL of 1 M glycine (pH 3.0) at room temperature for 30 min. This constituted the first round of biopanning.

The competitive targets were alternated in subsequent rounds: in the second round, wells were coated with AAV8 and AAV9, with AAV2 as the competitor; in the third round, wells were coated with AAV9 and AAV2, with AAV8 as the competitor; and in the fourth round, wells were coated with AAV8 and AAV2, with AAV9 as the competitor. Four iterative rounds yielded the final enriched phage population.

### 2.3. Phage ELISA

After completion of four biopanning cycles, 192 individual phage clones were randomly picked for binding-screening by ELISA. Wells of high-binding 96-well plates were coated with 5 × 10^8^ viral particles in PBS, incubated overnight at 4 °C, then washed three times with PBST and blocked with 200 µL of 3% BSA for 2 h at 37 °C.

Following a further three PBST washes, 100 µL of clarified phage supernatant was added to each well and incubated for 1 h at 37 °C. Plates were washed three times with PBST before the addition of 100 µL HRP-conjugated anti-M13 monoclonal antibody (diluted in PBST containing 1% BSA), followed by a 1 h incubation at 37 °C. After five stringent PBST washes, chromogenic development was initiated by adding 100 µL TMB substrate per well.

Reactions were stopped with 100 µL of 1 M H_2_SO_4_, and absorbance at 450 nm was measured using an Infinite® 200 (Tecan, Männedorf, Switzerland). Clones yielding OD_450_ values above the threshold of 0.5 were classified as positive. Positive clones were sequenced, and their VHH gene segments were aligned and annotated using the international ImMunoGeneTics information system (IMGT) (http://www.imgt.org, last accessed on 10 December 2024) for germline assignment.

### 2.4. VHHs Expression and Purification

The variable domain sequences encoding the VHHs, each C-terminal carrying an 8×His tag, were synthesized de novo (General Biol, Chuzhou, China) and subsequently cloned into the pTT5 mammalian expression vector using *Hind* III and *Bam* HI restriction sites. Recombinant plasmids were transfected into suspension-cultured 293F cells via transient transfection.

Following expression, 8×His-tagged VHHs were purified from the clarified cell supernatants using nickel-nitrilotriacetic acid (Ni-NTA) affinity chromatography. Elution was carried out using a linear gradient of imidazole (20 mM to 500 mM). The eluted fractions were dialyzed against PBS to remove imidazole, and protein purity was assessed via 12% SDS-PAGE under reducing conditions.

### 2.5. Affinity Measurements

To evaluate the binding specificity of VHHs, AAV2, AAV8, and AAV9 (1 × 10^9^ VP) were immobilized onto 96-well plates as the experimental group, while 3% BSA was used as a negative control. After blocking with 3% BSA and washing with PBST, purified VHHs were added and incubated for 1 h at 37 °C, followed by detection with an HRP-conjugated anti-His secondary antibody. Specific binding was determined by comparing signals from AAV-coated wells with those from BSA-coated controls.

For affinity determination, serial dilutions of VHHs (1 pM to 1000 nM) were applied under the same conditions. Absorbance at 450 nm was recorded, and binding curves were fitted using a four-parameter logistic model to calculate the half-maximal effective concentration (EC_50_) for each VHH.

### 2.6. Acid and Alkali Stability Assessment

100 μL of the test VHH was pre-incubated at room temperature for 48 h in either an alkaline solution (pH 13, containing 0.1 M NaOH) or an acidic solution (pH 2.0, containing 0.1 M glycine), followed by neutralization to physiological pH (pH 7.4). The binding activity of these VHH was then assessed by ELISA. The extent of VHH activity loss was calculated using the following formula:

$$\mathrm{Activity}\;\mathrm{Loss}\;\left(\%\right)=\frac{{\mathrm{OD}}_{450}\left(A\right)-{\mathrm{OD}}_{450}\left(B\right)}{{\mathrm{OD}}_{450}\left(A\right)}\times 100\%$$

where OD_450_(A) and OD_450_(B) represent the absorbance values at 450 nm of the positive control wells and the experimental wells, respectively.

### 2.7. Acid Elution Simulation Verification

AAV2, AAV8, AAV9 (1 × 10^9^ VP/well) and BSA (3% *w*/*v*) were coated onto high-binding 96-well plates and incubated overnight at 4 °C and then blocked. 100 μL of VHH solution (1000 nM) was added to each well and incubated at 37 °C for 1 h. Subsequently, 100 μL of acidic solutions at pH 2.0 or pH 3.0 (containing 0.1 M glycine) were applied to the wells for 5 min to induce elution. After washing with PBST, HRP-conjugated anti-His secondary antibody was added and incubated at 37 °C for 1 h. And then washing and measuring absorbance at 450 nm. Lower OD450 values indicate higher elution efficiency, as they reflect reduced amounts of bound target protein. The elution efficiency was calculated using the following formula:

$$\mathrm{Elution}\;\mathrm{Efficiency}\;(\%)=\frac{{\mathrm{OD}}_{450}\left(C\right)-{\mathrm{OD}}_{450}\left(D\right)}{{\mathrm{OD}}_{450}(C)}\times 100\%$$

where OD_450_(C) and OD_450_(D) represent the absorbance values of the positive control wells and acid-eluted wells, respectively.

### 2.8. VHH Coupling to CNBr-Activated Agarose Beads

The coupling process was performed using CNBr-activated Sepharose 4FF (YSK Bio, Zhuji, China), following the manufacturer’s instructions to generate the affinity purification resin “08”. A brief outline of the procedure is as follows:

Solution A (1 mM HCl, 0.5 M NaCl, pH 2.5): used for resin pretreatment to remove stabilizers; Solution B (0.2 M NaHCO_3_, 0.5 M NaCl, pH 8.3): VHH ligands were exchanged into this buffer and coupled to the resin at a density of 5–8 mg VHH/mL resin, with gentle mixing at 4 °C for 20 h; Solution C (0.1 M Tris-HCl, pH 8.0): used for washing and blocking unreacted active groups; Solution D (0.05 M Tris-HCl, 0.5 M NaCl, pH 8.5) and Solution E (0.1 M acetate, 0.5 M NaCl, pH 4.0): alternately applied three times to remove non-covalently bound ligands; Solution F (0.02 M PBS, 0.15 M NaCl, pH 7.4): used for the final wash, and the resin was stored in 20% ethanol. All solution exchanges are carried out in a glass filter funnel.

Ligand coupling was confirmed by BCA protein assay, in which the protein concentrations of the initial coupling solution and the wash fractions were compared to calculate the amount of ligand successfully immobilized.

### 2.9. AAV Production and Purification

AAV2, AAV8, and AAV9 were generated by triple-plasmid transient transfection in suspension-cultured 293F cells. Cells were seeded at 3 × 10^6^ viable cells/mL and transfected using linear polyethyleneimine (PEI MAX 40000, Polysciences Inc., Warrington, PA, USA pH 7.1) at a PEI: DNA mass ratio of 4:1. The plasmid ternary complex—comprising the vector (encoding enhanced green fluorescent protein, EGFP), Rep/Cap, and Ad helper plasmids—was delivered at an equimolar ratio (1:1:1). All plasmids used were supplied by Beijing Dingchi Biotechnology Co., Ltd. (Beijing, China).

At 24 h post-transfection, the culture was supplemented with R33 (final 2 g/L), NF606d-P1 (7.5% *v*/*v*), and NF606d-P2 (0.75% *v*/*v*). Cells were harvested by centrifugation 72 h post-transfection, and pellets were resuspended in cold PBS for lysis under nuclease treatment: incubated with 0.5% Tween-20, 20 U/mL Benzonase (Merck, Darmstadt, Germany), and 1 mM MgCl_2_ for 3 h at 37 °C under orbital agitation. After clarification by centrifugation at 12,000× *g* for 30 min at 4 °C, the supernatant was sterile-filtered through 0.8 µm polyether sulfone (PES) membranes (MilliporeSigma, Burlington, MA, USA).

Affinity resin was packed into 1 mL chromatography columns and integrated into an ÄKTA pure 25 system (Cytiva, Marlborough, MA, USA). Columns were equilibrated with 20 column volumes (CV) of PBS, pH 7.4. AAV lysates were loaded at 1 mL/min (0.66 cm/min linear velocity), followed by 10 CV PBS washing. Bound virions were acid-eluted with 0.1 M glycine (pH 2.0). Post-elution, the resin was regenerated with 15 CV of 0.1 M NaOH (1 mL/min flow rate; 15 min total contact time) and stored in 20% (*v*/*v*) ethanol. For comparison, we also employed a commercial multi-serotype affinity resin (with binding specificity for AAV2, AAV5, AAV8, and AAV9). The same purification workflow was applied, whereas column cleaning and regeneration were performed according to the manufacturer’s recommended protocol. Purified AAV was immediately neutralized with 2 M Tris (pH 9.0) prior to immunoblot analysis.

### 2.10. AAV Infection and Transduction Assays

293T cells were seeded at a density of 1 × 10^5^ cells per well in 48-well plates containing 400 μL of DMEM containing 10% heat-inactivated fetal bovine serum (FBS) (Gibco, Thermo Fisher Scientific, Waltham, MA, USA). Purified AAV was added at a multiplicity of infection (MOI) of 10^5^ viral genomes (VG) per cell. After 24 h of incubation at 37 °C, cells were washed twice with PBS to remove unbound virions, fresh medium was added, and fluorescence reporter expression was imaged under identical exposure settings using a Leica fluorescence microscope (Leica Microsystems GmbH, Wetzlar, Germany). For quantitative analysis of transduction efficiency, flow cytometry was performed to determine the percentage of EGFP-positive cells. Briefly, cells were processed under the same experimental conditions, harvested with trypsin at the end of transduction, washed twice with PBS, and resuspended in 200 μL of PBS. Flow cytometric analysis was carried out using a BD FACSAria™ III (BD Biosciences, San Jose, CA, USA), and data were analyzed with FlowJo™ v10.8.1 software (FlowJo, LLC, Ashland, OR, USA).

### 2.11. Prediction of Potential Binding Sites Between VHH and AAV2, AAV8, and AAV9

The three-dimensional structure of the VHH was modeled using the SWISS-MODEL server (https://swissmodel.expasy.org/, last accessed on 10 March 2025), and the resulting PDB files were downloaded. The antigen sequences corresponding to AAV capsid proteins were obtained from UniProt with accession numbers P03135 (AAV2), Q8JQF8 (AAV8), and Q6JC40 (AAV9). Protein-protein docking was performed using the HDOCK server (http://hdock.phys.hust.edu.cn/, last accessed on 28 March 2025). Predicted VHH–capsid binding interfaces were further analyzed using the PDBePISA (https://www.ebi.ac.uk/pdbe/pisa/, last accessed on 15 May 2025). Docking results were visualized and annotated using PyMOL (Schrödinger, LLC, New York, NY, USA).

### 2.12. Statistical Analysis

All experiments were replicated three times, and representative data or pooled data from repeat experiments were recorded. Statistical analyses were conducted using GraphPad Prism 10.3 (Dotmatics, San Diego, CA, USA). Two tailed unpaired t-tests were used to compare results between two groups. *p* < 0.05 was considered to indicate a statistically significant difference. Exact *p* values and biological replicates (N) are reported in Figure legends.

## 3. Results

### 3.1. Selection and Characterization of VHHs Targeting AAV2, AAV8, and AAV9

**Figure 1:**
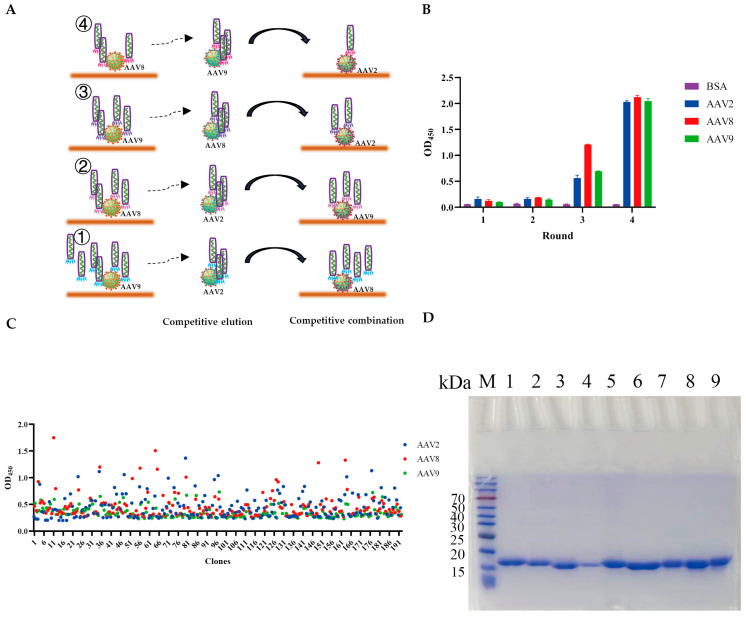
Selection and expression of VHHs against AAV2, AAV8, and AAV9. (**A**) Competitive biopanning strategy diagram. ① Round 1: Screening was performed in AAV9-coated wells, followed by competition with AAV2. The phages eluted by this competition were subsequently transferred to AAV8-coated wells for incubation. ② Round 2: Screening was carried out in AAV8- and AAV9-coated wells, with AAV2 used as the competitive eluent. ③ Round 3: Screening was performed in AAV9- and AAV2-coated wells, with AAV8 as the competitive eluent. ④ Round 4: Screening was carried out in AAV8- and AAV2-coated wells, with AAV9 as the competitive eluent. (**B**) Enrichment of phage particles displaying VHHs specific to AAV2, AAV8, and AAV9 over four rounds of biopanning. (**C**) ELISA screening of 192 phage supernatants for identification of positive clones. (**D**) SDS-PAGE analysis of VHHs specific to AAV2, AAV8, and AAV9. M: protein marker. Lanes 1–9: VHH8, VHH22, VHH69, VHH113, VHH38, VHH44, VHH68, VHH131, and VHH95.

To enrich VHHs with cross-serotype binding activity, a competitive biopanning strategy was employed using AAV2, AAV8, and AAV9 capsids alternately as immobilized antigens or soluble competitors. As AAV8 and AAV9 are closely related in sequence, they were prioritized in the early rounds, where AAV8/9 was immobilized and soluble AAV2 was used for competitive elution with recovery on another serotype plate. In later rounds, AAV8 and AAV9 alternated as antigen/competitor, and displaced phages were recaptured on AAV2-coated plates, yielding VHHs that retained AAV8/9 cross-reactivity while also acquiring AAV2 binding (Figure 1A).

After four rounds of selection, the phage pool exhibited markedly enhanced binding toward AAV2, AAV8, and AAV9, showing approximately a fourfold increase compared with the first-round pool (Figure 1B). 192 individual clones were randomly selected and induced to produce monoclonal phage supernatants. Clones were considered positive if their absorbance values (OD) for all three serotypes, AAV2, AAV8, and AAV9, exceeded 0.5 in antigen-coated wells, and were subsequently subjected to DNA sequencing (Figure 1C). Sequence analysis of the positive clones using IMGT revealed nine distinct VHH sequences. Sequence variability was predominantly confined to complementarity-determining region 3 (CDR3), with additional heterogeneity observed in CDR1, CDR2, and the framework regions (FRs).

### 3.2. Expression and Purification of VHHs

All candidate VHHs were engineered with a C-terminal 8×His tag for purification. VHH genes were cloned into the pTT5 expression vector and transiently transfected into suspension-cultured 293F cells. Culture supernatants were collected on day 7 and subjected to immobilized metal affinity chromatography (IMAC) using Ni-NTA resin. SDS-PAGE followed by Coomassie blue staining confirmed the successful expression of recombinant VHHs, yielding distinct monomeric bands with molecular weights of 15–20 kDa and a purity exceeding 95% (Figure 1D).

### 3.3. Binding Specificity and Affinity of the VHHs

**Figure 2:**
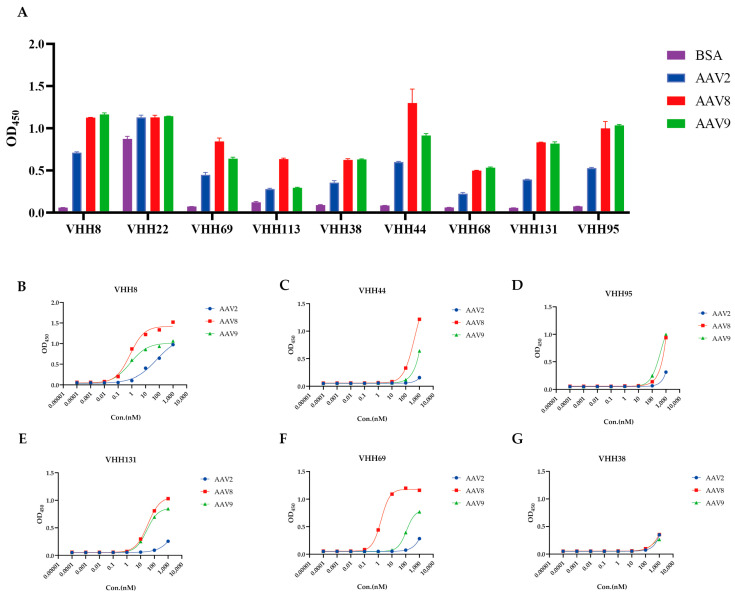
Specificity and binding affinity of purified VHHs. (**A**) ELISA analysis of the binding specificity of purified VHHs to AAV2, AAV8, and AAV9. (**B**–**G**) ELISA-based evaluation of the binding affinity of purified VHHs to AAV2, AAV8, and AAV9. VHH22, VHH113, and VHH68 were excluded from this analysis due to their suboptimal binding specificity.

Among the nine clones, VHH22 exhibited nonspecific binding, VHH68 displayed weak reactivity with AAV2, and VHH113 showed strongly binding to AAV8. In contrast, the remaining six VHH clones demonstrated robust and specific binding to all three AAV serotypes—AAV2, AAV8, and AAV9 (Figure 2A).

The binding affinities of these six VHHs were further quantified by ELISA, yielding distinct half-maximal effective concentration (EC_50_) values against each serotype (Table 1; Figure 2B–G). Notably, clone VHH8 exhibited high-affinity, cross-serotype binding, with EC_50_ values of 64.78 nM for AAV2, 0.7559 nM for AAV8, and 0.7733 nM for AAV9.

### 3.4. Stability Assessment of VHH8

**Figure 3:**
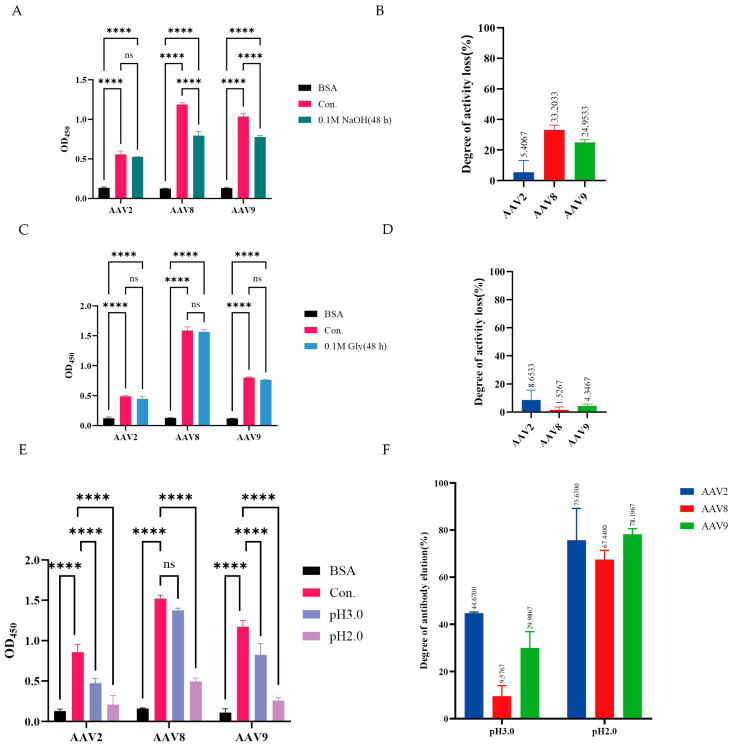
Acid and alkaline stability and purification potential of VHH8 (**A**) Assessment of the alkaline stability of VHH8 by ELISA. (**B**) Activity loss rate of VHH8 after treatment with 0.1 M NaOH for 48 h. (**C**) Assessment of the acid resistance of VHH8 by ELISA. (**D**) Activity loss rate of VHH8 after treatment with 0.1 M glycine (Gly) for 48 h. (**E**) ELISA-based evaluation of AAV2, AAV8, and AAV9 elution from VHH8 under pH 2.0 and pH 3.0 conditions. (**F**) Elution efficiency of AAV2, AAV8, and AAV9 from VHH8 at pH 2.0 and pH 3.0, as determined by ELISA. n = 3, ns: not significant, *p* > 0.05; **** *p* < 0.0001.

Given its cross-serotype reactivity, the stability of VHH8 under conditions mimicking affinity chromatography was evaluated. VHH8 (1000 nM) was incubated for 48 h under alkaline (pH 13.0) or acidic (pH 2.0) conditions, and binding activity to AAV2, AAV8, and AAV9 was assessed by ELISA (Figure 3A,C). After alkaline treatment, the binding activity of VHH8 toward AAV2, AAV8, and AAV9 decreased by 5.41%, 33.20%, and 24.95%, respectively (Figure 3B), whereas acidic treatment reduced the binding activity by 8.65%, 1.53%, and 4.35%, respectively (Figure 3D). These results demonstrate that VHH8 retains remarkable stability under extreme pH conditions.

Under pH 3.0 conditions, a moderate level of elution was observed, while effective elution occurred at pH 2.0 (Figure 3E). At pH 3.0, elution efficiencies were 44.67% for AAV2, 9.57% for AAV8, and 29.98% for AAV9, whereas pH 2.0 resulted in substantially higher values of 75.63%, 67.44%, and 78.20%, respectively (Figure 3F). These findings demonstrate that VHH8 exhibits efficient acid-induced release while retaining strong pH stability, highlighting its potential as a robust affinity ligand for cross-serotype AAV purification.

### 3.5. Affinity Purification of AAV with VHH8-Based Chromatography Resin

**Figure 4:**
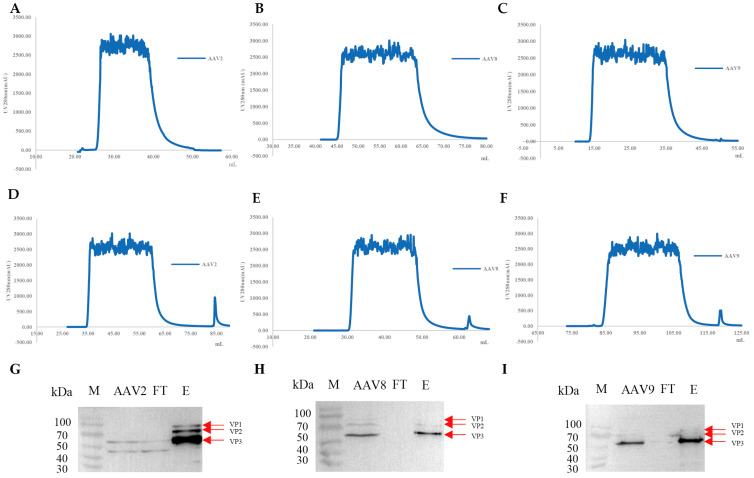
Validation of the purification capability of VHH8−coupled resin (08 resin). (**A**–**C**) Affinity purification chromatogram of AAV2, AAV8, and AAV9 using commercially available resin on the AKTA Pure system. (**D**–**F**) Affinity purification chromatogram of AAV2, AAV8, and AAV9 using 08 resin on the AKTA Pure system. (**G**–**I**) Western blot analysis of AAV2, AAV8, and AAV9 purified by 08 resin. Samples included the AAV2/8/9 (from cell lysate), flow-through (FT), and eluted fractions (E).

Following the conjugation process, the VHH concentrations before and after coupling were determined, confirming that efficient conjugation had been achieved (Figure S1). Clarified lysates of AAV2, AAV8, and AAV9 were applied to both a commercial affinity resin (with binding capabilities for the target serotypes) and our VHH8-coupled resin. While the commercial resin showed no detectable elution peaks (Figure 4A–C), the VHH8-coupled resin generated clear, well-resolved peaks for all three serotypes (Figure 4D–F), confirming its efficient capture and acid-elution performance. Western blot analysis further confirmed the presence of VP1, VP2, and VP3 in the eluted fractions from our VHH8-coupled resin (Figure 4G–I). No discernible purification peak was observed with the commercial control resin; therefore, no eluate was collected for further analysis.

### 3.6. Preservation of In Vitro Bioactivity of AAV Serotypes Following Purification

**Figure 5:**
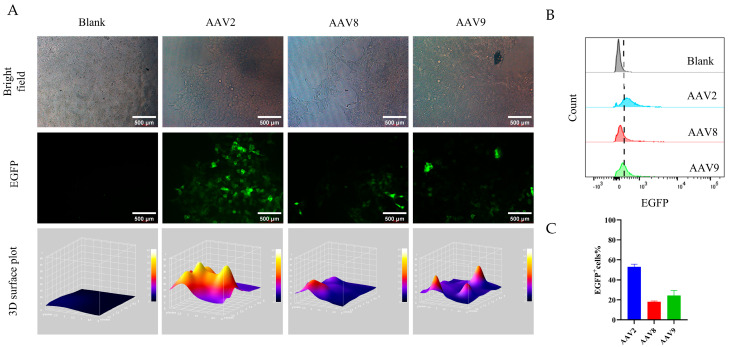
In vitro bioactivity of AAV2, AAV8, and AAV9 purified using 08 resin in 293T cells. (**A**) Fluorescence image of purified AAV infected 293T cells. Shown are representative bright−field images, EGFP fluorescence images, and corresponding 3D surface plots. Columns correspond to the Blank control, AAV2, AAV8, and AAV9 transduced groups. (**B**) Flow cytometry histograms showing fluorescence intensity shifts in 293T cells infected with purified AAV2, AAV8, and AAV9 at 37 °C for 24 h. (**C**) Quantification of transduction efficiency, presented as the percentage of EGFP^+^ 293T cells derived from the histogram analysis in panel (**B**).

To evaluate the in vitro bioactivity of AAV serotypes purified using VHH8-based affinity chromatography, 293T cells were infected at a multiplicity of infection (MOI) of 10^5^ viral genomes (vg) per cell. At 24 h post-infection, reporter gene expression was assessed by fluorescence microscopy. All three purified AAV serotypes demonstrated efficient transduction in 293T cells, indicating that the VHH8-based purification process preserved viral infectivity (Figure 5A). In addition, flow cytometry was performed under the same conditions to quantitatively assess transduction efficiency by measuring reporter gene expression. The results showed that AAV2 exhibited the highest transduction efficiency, exceeding 50%, whereas AAV8 and AAV9 displayed efficiencies of approximately 20% (Figure 5B,C).

### 3.7. Prediction of the VHH8 Binding Epitope on the AAV Capsid

**Figure 6:**
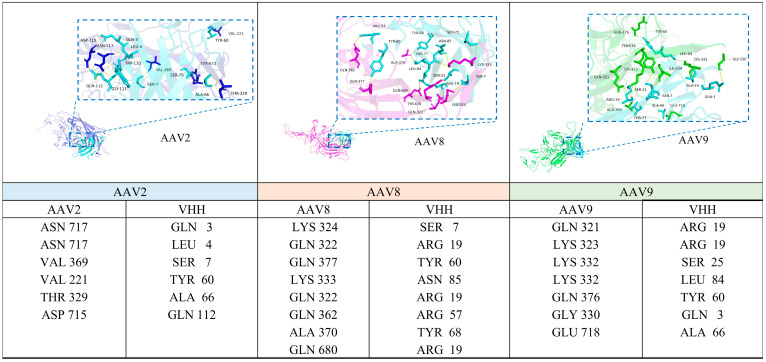
Predicted interactions between VHH8 and AAV2/8/9 VP3 proteins. The predicted binding interfaces between VHH8 (cyan) and VP3 proteins of AAV2 (blue), AAV8 (pink), and AAV9 (green) are shown. Binding sites are highlighted with blue boxes, and the interactions are represented as hydrogen bonds (yellow dashed lines). Specific amino acid residues involved in the interactions are listed below each corresponding panel.

Given that the VP3 subunit constitutes approximately 80% of the assembled AAV capsid and is highly surface-exposed, our analysis focused on the interaction between VHH8 and VP3. After modeling and molecular docking, PyMOL was used for structural visualization, focusing on hydrogen bonds at the VHH-antigen interface (Figure 6). Hydrogen bond analysis revealed multiple contacts between the VHH and each AAV serotype. Several VHH residues were involved in interactions across all three complexes. TYR60 was consistently observed to form hydrogen bonds with the capsid in all serotypes. ARG19 formed hydrogen bonds with the capsid in both AAV8 and AAV9 complexes. Additional interface residues found in two or more serotypes included SER7, GLN3, and ALA66.

The viral VP3 residues that interact with AAV were identified as THR329 and ASN717 in AAV2, GLN322 and LYS333 in AAV8, and GLY330 and LYS332 in AAV9. These residues are located on surface-exposed regions of the capsid protein, primarily within or adjacent to the variable region II (VR-II), which corresponds to the DE loop. The DE loop is a prominent structural feature of the AAV capsid known to participate in surface interactions. Structural mapping showed that most of the identified interface residues fall within the DE loop region or its immediate surroundings.

Collectively, these results indicate that the VHH binds in a region near the DE loop across all three AAV serotypes. The overlapping sets of interacting residues suggest a structurally similar binding mode among AAV2, AAV8, and AAV9.

## 4. Discussion

Adeno-associated virus (AAV) is the most widely used vector in gene therapy, with over 200 related clinical trials registered globally [2]. As the industrialization of AAV-based gene therapies accelerates, the development of efficient, reusable, and production-compatible affinity purification strategies has become critical for ensuring product quality and reducing manufacturing costs. This study introduces a competitive elution strategy into the phage display platform, successfully identifying a VHH with the ability to bind to AAV2/8/9 and exhibit significant alkaline stability. This dual functionality offers an innovative solution for large-scale, GMP-compliant purification processes, potentially extending resin lifespan, improving cost-effectiveness, and enabling its application across different AAV serotypes in industrial production.

Traditional phage display screening typically targets a single AAV serotype, resulting in high-affinity ligands that are often serotype-restricted. To overcome this limitation, this study employed AAV2, AAV8, and AAV9 as competitive targets, directing the selection pressure towards conserved regions of the viral capsid. This approach successfully identified VHHs with cross-serotype binding capability. Compared to conventional methods, this strategy not only shortened the screening timeline but also yielded ligands with broader binding properties, thereby enhancing compatibility and flexibility for downstream applications. Molecular modeling further revealed that the selected VHH predominantly recognizes the DE-loop region of the AAV VP3 protein. This region is a structurally stable and relatively conserved epitope within the capsid, and it is also a key functional loop involved in host receptor binding and neutralizing antibody recognition [30]. By targeting these conserved regions, the ligands selected through the competitive screening strategy can bind to AAV2, AAV8, and AAV9 serotypes. This finding molecularly explains the advantage of the competitive selection method, where functional, conserved binding sites are prioritized to obtain robust affinity ligands.

The VHH selected in this study demonstrated excellent stability, maintaining their structural integrity and binding activity even in 0.1 M NaOH. This feature allows them to meet the alkaline cleaning-in-place (CIP) requirements for affinity chromatography resins in practical production, significantly extending resin lifespan and reducing production costs. Notably, the competitive screening strategy not only improved binding capacity but also likely helped to enrich more alkali-resistant ligands, offering new possibilities for developing reusable affinity matrices.

While the selected VHHs exhibited ideal binding capacity and alkaline stability, there is still room for improvement in purification recovery rates. Recovery data were not presented in this study, as the experimental system is still in the early validation phase and has not undergone full process optimization. The lower recovery rates may be attributed to several factors: (1) Differences in binding affinity of VHHs for different serotypes; (2) Suboptimal immobilization methods and spatial orientation, which limit the accessibility of effective binding sites; (3) The need for further optimization of support materials and elution conditions. The immediate future work will therefore focus on systematically optimizing the coupling chemistry to determine the optimal ligand density that maximizes the dynamic binding capacity. Subsequently, the operational stability of the resin will be rigorously challenged through extensive CIP cycles with 0.1 M NaOH. Success in these areas will address the recovery yield challenge and establish the full commercial potential of this affinity ligand.

Overall, the competitive selection strategy proposed in this study not only theoretically demonstrates the feasibility of cross-serotype binding through targeting conserved regions but also successfully yields alkali-resistant VHHs—a high-value trait. Despite current limitations such as low purification recovery, this platform lays a strong foundation for the further optimization and application of affinity ligands. The selected VHHs show strong affinity for AAV2, AAV8, and AAV9, highlighting their potential as ligands for AAV serotypes. Although biological activity was retained in the purified viruses, a direct comparison of transduction efficiency between this method and standard purification approaches will be included in future work to more comprehensively evaluate the relative effectiveness of the strategy. In addition, mutagenesis analysis will be performed to experimentally validate the predicted binding epitopes, thereby enhancing the reliability and functional interpretation of the structural model and advancing AAV industrial production as well as affinity ligand development.

## 5. Conclusions

In this study, a competitive biopanning strategy was employed to isolate a single-domain antibody (VHH) capable of recognizing AAV2, AAV8, and AAV9. The VHH retained activity after 0.1 M NaOH treatment, an available concentration used in CIP procedures for chromatography media [31], thereby meeting cleaning requirements. Its cross-serotype adaptability facilitates downstream process standardization, offering a novel solution for safe and efficient industrial-scale AAV production.

## Figures and Tables

**Table 1:** VHHs against AAV2/8/9—EC_50_.

Name	EC_50_ (nM)			R^2^		
	AAV2	AAV8	AAV9	AAV2	AAV8	AAV9
VHH8	64.78	0.7559	0.7733	0.9919	0.9910	0.9941
VHH69	>1000	1.615	114.1	1.000	0.9996	1.000
VHH38	>1000	>1000	378.4	1.000	1.000	1.000
VHH44	>1000	433.5	>1000	0.9920	1.000	1.000
VHH131	>1000	32.96	28.55	0.9999	0.9998	0.9999
VHH95	>1000	>1000	421.6	0.9996	1.000	1.000

## Data Availability

All datasets and reagents generated through this study are available from the corresponding author.

## Supplementary Materials

The following supporting information can be downloaded at: https://www.mdpi.com/article/10.3390/v17101289/s1, Figure S1: VHH concentration before and after coupling.

## References

[1] Pupo A., Fernández A., Low S.H., François A., Suárez-Amarán L., Samulski R.J. (2022). AAV Vectors: The Rubik’s Cube of Human Gene Therapy. Mol. Ther..

[2] Naso M.F., Tomkowicz B., Perry W.L., Strohl W.R. (2017). Adeno-Associated Virus (AAV) as a Vector for Gene Therapy. Biodrugs.

[3] Suarez-Amaran L., Song L., Tretiakova A.P., Mikhail S.A., Samulski R.J. (2025). AAV Vector Development, Back to the Future. Mol. Ther. J. Am. Soc. Gene Ther..

[4] Vandenberghe L.H., Wilson J.M., Gao G. (2009). Tailoring the AAV Vector Capsid for Gene Therapy. Gene Ther..

[5] Russell S., Bennett J., Wellman J.A., Chung D.C., Yu Z.F., Tillman A., Wittes J., Pappas J., Elci O., McCague S. (2017). Efficacy and Safety of Voretigene Neparvovec (AAV2-hRPE65v2) in Patients with RPE65-Mediated Inherited Retinal Dystrophy: A Randomised, Controlled, Open-Label, Phase 3 Trial. Lancet.

[6] Mendell J.R., Al-Zaidy S., Shell R., Arnold W.D., Rodino-Klapac L.R., Prior T.W., Lowes L., Alfano L., Berry K., Church K. (2017). Single-Dose Gene-Replacement Therapy for Spinal Muscular Atrophy. N. Engl. J. Med..

[7] Wang J.-H., Gessler D.J., Zhan W., Gallagher T.L., Gao G. (2024). Adeno-Associated Virus as a Delivery Vector for Gene Therapy of Human Diseases. Signal Transduct. Target. Ther..

[8] Ozelo M.C., Mahlangu J., Pasi K.J., Giermasz A., Leavitt A.D., Laffan M., Symington E., Quon D.V., Wang J.-D., Peerlinck K. (2022). Valoctocogene Roxaparvovec Gene Therapy for Hemophilia A. N. Engl. J. Med..

[9] Li C., Samulski R.J. (2020). Engineering Adeno-Associated Virus Vectors for Gene Therapy. Nat. Rev. Genet..

[10] Pipe S.W., Leebeek F.W.G., Recht M., Key N.S., Castaman G., Miesbach W., Lattimore S., Peerlinck K., Van der Valk P., Coppens M. (2023). Gene Therapy with Etranacogene Dezaparvovec for Hemophilia B. N. Engl. J. Med..

[11] Grimm D., Lee J.S., Wang L., Desai T., Akache B., Storm T.A., Kay M.A. (2008). In Vitro and In Vivo Gene Therapy Vector Evolution via Multispecies Interbreeding and Retargeting of Adeno-Associated Viruses. J. Virol..

[12] Chauhan M., Daugherty A.L., Khadir F., Duzenli O.F., Hoffman A., Tinklenberg J.A., Kang P.B., Aslanidi G., Pacak C.A. (2024). AAV-DJ Is Superior to AAV9 for Targeting Brain and Spinal Cord, and de-Targeting Liver across Multiple Delivery Routes in Mice. J. Transl. Med..

[13] Pietersz K.L., Plessis F.D., Pouw S.M., Liefhebber J.M., van Deventer S.J., Martens G.J.M., Konstantinova P.S., Blits B. (2021). PhP.B Enhanced Adeno-Associated Virus Mediated-Expression Following Systemic Delivery or Direct Brain Administration. Front. Bioeng. Biotechnol..

[14] Kim J.J., Kurial S.N., Choksi P.K., Nunez M., Lunow-Luke T., Bartel J., Driscoll J., Her C.L., Dhillon S., Yue W. (2025). AAV Capsid Prioritization in Normal and Steatotic Human Livers Maintained by Machine Perfusion. Nat. Biotechnol..

[15] Cabanes-Creus M., Navarro R.G., Liao S.H.Y., Scott S., Carlessi R., Roca-Pinilla R., Knight M., Baltazar G., Zhu E., Jones M. (2023). Characterization of the Humanized FRG Mouse Model and Development of an AAV-LK03 Variant with Improved Liver Lobular Biodistribution. Mol. Ther. Methods Clin. Dev..

[16] Zhao Y., Zhang L., Wang D., Chen B., Shu Y. (2022). Approaches and Vectors for Efficient Cochlear Gene Transfer in Adult Mouse Models. Biomolecules.

[17] Schwartz M.K., Likhite S., Vetter T.A., Baird M.C., McGovern V., Sierra Delgado A., Mendel T., Burghes A., Meyer K.C. (2023). In-Depth Comparison of Anc80L65 and AAV9 Retinal Targeting and Characterization of Cross-Reactivity to Multiple AAV Serotypes in Humans. Mol. Ther. Methods Clin. Dev..

[18] Mnyandu N.Z., Limani S.W., Ely A., Wadee R., Arbuthnot P., Maepa M.B. (2025). Long-Term Inhibition of Hepatitis B Virus Gene Expression by a Primary Microrna Expressing Ancestral Adeno-Associated Viral Vector. Virol. J..

[19] Khabou H., Desrosiers M., Winckler C., Fouquet S., Auregan G., Bemelmans A.P., Sahel J.A., Dalkara D. (2016). Insight into the Mechanisms of Enhanced Retinal Transduction by the Engineered AAV2 Capsid Variant -7m8. Biotechnol. Bioeng..

[20] Mietzsch M., Smith J.K., Yu J.C., Banala V., Emmanuel S.N., Jose A., Chipman P., Bhattacharya N., McKenna R., Agbandje-McKenna M. (2020). Characterization of AAV-Specific Affinity Ligands: Consequences for Vector Purification and Development Strategies. Mol. Ther. Methods Clin. Dev..

[21] Florea M., Nicolaou F., Pacouret S., Zinn E.M., Sanmiguel J., Andres-Mateos E., Unzu C., Wagers A.J., Vandenberghe L.H. (2023). High-Efficiency Purification of Divergent AAV Serotypes Using AAVX Affinity Chromatography. Mol. Ther. Methods Clin. Dev..

[22] Wang Q., Lock M., Prongay A.J., Alvira M.R., Petkov B., Wilson J.M. (2015). Identification of an Adeno-Associated Virus Binding Epitope for AVB Sepharose Affinity Resin. Mol. Ther. Methods Clin. Dev..

[23] Chu W., Shastry S., Barbieri E., Prodromou R., Greback-Clarke P., Smith W., Moore B., Kilgore R., Cummings C., Pancorbo J. (2023). Peptide Ligands for the Affinity Purification of Adeno-Associated Viruses from HEK 293 Cell Lysates. Biotechnol. Bioeng..

[24] Shastry S., Chu W., Barbieri E., Greback-Clarke P., Smith W.K., Cummings C., Minzoni A., Pancorbo J., Gilleskie G., Ritola K. (2024). Rational Design and Experimental Evaluation of Peptide Ligands for the Purification of Adeno-Associated Viruses via Affinity Chromatography. Biotechnol. J..

[25] van Lieshout L.P., Stegelmeier A.A., Rindler T.N., Lawder J.J., Sorensen D.L., Frost K.L., Booth S.A., Bridges J.P., Wootton S.K. (2023). Engineered AAV8 Capsid Acquires Heparin and AVB Sepharose Binding Capacity but Has Altered in Vivo Transduction Efficiency. Gene Ther..

[26] Jin B., Odongo S., Radwanska M., Magez S. (2023). NANOBODIES®: A Review of Generation, Diagnostics and Therapeutics. Int. J. Mol. Sci..

[27] Klooster R., Maassen B.T.H., Stam J.C., Hermans P.W., Ten Haaft M.R., Detmers F.J.M., de Haard H.J., Post J.A., Theo Verrips C. (2007). Improved Anti-IgG and HSA Affinity Ligands: Clinical Application of VHH Antibody Technology. J. Immunol. Methods.

[28] Zhang L., Zang B., Huang C., Ren J., Jia L. (2019). One-Step Preparation of a VHH-Based Immunoadsorbent for the Extracorporeal Removal of Β2-Microglobulin. Molecules.

[29] Issa S.S., Shaimardanova A.A., Solovyeva V.V., Rizvanov A.A. (2023). Various AAV Serotypes and Their Applications in Gene Therapy: An Overview. Cells.

[30] Zhang R., Xu G., Cao L., Sun Z., He Y., Cui M., Sun Y., Li S., Li H., Qin L. (2019). Divergent Engagements between Adeno-Associated Viruses with Their Cellular Receptor AAVR. Nat. Commun..

[31] Grönberg A., Eriksson M., Ersoy M., Johansson H.J. (2011). A Tool for Increasing the Lifetime of Chromatography Resins. MAbs.

