# Supplementary material for: Screening of Single-Domain Antibodies to Adeno-Associated Viruses with Cross-Serotype Specificity and a Wide pH Tolerance
Source: Viruses. 2025 Sep 23;17(10):1289. doi: 10.3390/v17101289 (PMC12568183; doi:10.3390/v17101289)
Supplement: Supplementary file 1 [file viruses-17-01289-s001.zip › viruses-3865185-supplementary.pdf]

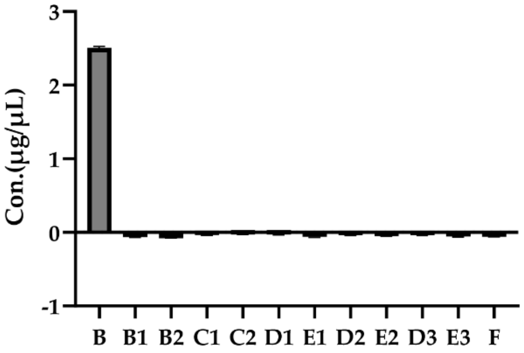

Figure S1: VHH concentration before and after coupling. B: initial VHH solution; B1: solution after completion of coupling; B2: wash solution with Solution B; C1: solution after washes with Solution C; C2: solution after blocking with Solution C; D1–E3: solutions obtained from three alternating washes with Solution D and Solution E; F: solution after washing with Solution F. n = 3.
